# Sub-neutralizing levels of antibodies against RSV F protein enhance RSV infection via Fc-FcγR interactions

**DOI:** 10.3389/fimmu.2025.1594937

**Published:** 2025-06-10

**Authors:** Tingting Luo, Xin Zhao, Yi Lu, Wen Tan, Bingming Peng, Li Zhong, Jinhua Ma, Chenghao Mei, Huaqin Bu, Daiyin Tian

**Affiliations:** Department of Respiratory Medicine, Children’s Hospital of Chongqing Medical University, National Clinical Research Center for Child Health and Disorders, Ministry of Education Key Laboratory of Child Development and Disorders, China International Science and Technology Cooperation base of Child Development and Critical Disorders, Chongqing Key Laboratory of Child Rare Diseases in Infection and Immunity, Chongqing, China

**Keywords:** respiratory syncytial virus, nirsevimab, antibody-dependent enhancement, Fc gamma receptor, viral lifecycle

## Abstract

Respiratory syncytial virus (RSV) is a major cause of severe lower respiratory tract infections in infants, and antibody-dependent enhancement (ADE) presents a significant challenge in vaccine and antibody development. This study investigated the molecular mechanism of ADE mediated by sub-neutralizing antibodies against RSV F protein. We demonstrated that anti-RSV F antibody could facilitate RSV attachment and endocytosis into cells via Fc-Fc gamma receptor (FcγR) interactions while only at sub-neutralizing levels, it increased RSV replication and release, thereby inducing ADE. ADE was abrogated following the secondary occlusion of F protein using nirsevimab Fab fragments. During ADE, intracellular inflammatory signaling pathways were overactivated, resulting in elevated secretion of pro-inflammatory factors (IL-1β, IL-6, TNF-α). FcγRI was identified as the dominant receptor of ADE through blockade experiments, and dual inhibition of FcγRI and TLR4 abolished ADE, highlighting their synergistic role. Furthermore, ADE-induced cytokines upregulated FcγRs expression, creating a self-reinforcing inflammatory loop. Overall, our findings indicated that (1) sub-neutralizing F-specific antibodies increase RSV entry via FcγRs, whereby partially unsealed F protein allows subsequent membrane fusion and enhances replication; (2) RSV ADE pathogenesis involves the coordination of FcγRI and TLR4 signaling pathways, and is amplified by cytokine-driven upregulation of FcγRs. These findings are helpful for the development of next-generation antibody and vaccine against RSV.

## 1 Introduction

Human respiratory syncytial virus (RSV) is an enveloped, single-stranded RNA virus belonging to the *Paramyxoviridae* family. It is the leading cause of lower respiratory tract infections in children under 5 years of age, with severe infections often occurring in infants younger than 6 months (1). This poses a significant public health and socio-economic burden globally. Current effective prevention strategies for RSV infection in infants and children include monoclonal antibodies and maternal vaccination to confer transplacental neutralizing antibodies (2–4). Passive immunization with monoclonal antibodies includes palivizumab, which is approved for use in infants at high risk of RSV disease, and nirsevimab, which is approved for use in all infants by the European Medicines Agency (EMA) in November 2022 (4–7). At present, vaccines for active immunization against RSV in children are under development.

Alarmingly, July 2024 witnessed severe RSV cases in a Phase I trial evaluating Moderna’s mRNA-1345 and mRNA-1365 vaccine candidates in vaccinated infants (8). Within the cohort of participants aged 5 to <8 months, 5 vaccinated infants (12.5% of participants) developed clinically significant severe/very severe RSV versus 1 case (5% of participants) in placebo controls, with elevated rates of disease progression in vaccinees. This phenomenon may be attributed to enhanced respiratory disease (ERD). Historically, ERD was documented in the 1960s among infants vaccinated with formalin-inactivated RSV (FI-RSV) (9). Of the 31 infants who received FI-RSV, 25 required hospitalization and even 2 fatalities were recorded.

ERD involves a variety of molecular mechanisms, including antibody-mediated processes such as Fc receptor-driven effector functions and complement system activation (that is antibody-dependent enhancement, ADE) (10). Studies have reported that the neonatal Fc receptor (FcRn) can enhance HIV and cytomegalovirus transcytosis to promote infection (11–13). Research on mice found that FI-RSV-induced non-neutralizing IgG antibodies form virus-antibody complexes in the lung, triggering complement activation and subsequent tissue damage (14). ADE occurs when sub-neutralizing or non-neutralizing antibodies facilitate viral entry into Fc gamma receptor (FcγR)-expressing cells rather than conferring protection (10, 15, 16), a mechanism well-documented in dengue, Ebola, and influenza infections (17–20). Additionally, *in vitro* evidences confirm antibodies against RSV F protein enhance viral infection in FcγRs-bearing cells, such as monocytes/macrophages and NK cells (21–23). A study on FI-RSV immunization-induced enhanced respiratory disease in bonnet monkeys revealed increased RSV replication within perivascular mononuclear cells in the lungs (24). FcγRs can induce a range of effects, including antibody-dependent cellular phagocytosis (ADCP), antibody-dependent cellular cytotoxicity (ADCC) and inflammatory mediator production. However, the underlying mechanism of FcγR-mediated RSV ADE remains poorly understood.

To investigate the mechanisms underlying RSV ADE, we explored the life cycle of RSV and assessed molecular alterations in cells in the presence of antibodies to RSV F protein. Additionally, we conducted a preliminary analysis of the involvement of FcγRs in ADE. Our study may provide valuable insights for the development of RSV vaccines and antibodies aiming at mitigating the risk of ADE.

## 2 Materials and methods

### 2.1 Cells and virus

HEp-2 cell lines were gifted by Enmei Liu’s group from Children’s Hospital of Chongqing Medical University (Chongqing, China), and were maintained in DMEM containing 10% heat-inactivated FBS. THP-1 cell lines were purchased from Procell Life Science & Technology Co.,Ltd (Wuhan, China), and were maintained in RPMI 1640 medium containing 10% FBS.

RSV A2 was gifted by Enmei Liu’s group and recombinant red fluorescent protein (RFP)-expressing RSV A2 (rrRSV) was provided by Yuqing Wang’s group from Children’s Hospital of Suzhou Medical University (Suzhou, China). Both RSV A2 and rrRSV were grown on HEp-2 cells, purified via sucrose gradient ultracentrifugation, and stored at -80°C until use. Virus titers were determined by plaque assays.

### 2.2 RSV infection

Nirsevimab, nirsevimab Fab, and palivizumab, provided by TRINOMAB BIOTECH CO., LTD. (Zhuhai, China), were serially diluted (10-fold gradient) from original concentrations (1 µg/µL). RSV A2 was incubated with these antibody dilutions at 37°C for 1 h. Subsequently, HEp-2 cells (MOI=1) and THP-1 cells (MOI=10) were infected and incubated at 37°C for 1 h. Afterward, the medium was replaced, and the cells continued to incubate at 5% CO2, 37°C for 24 h. These cells were then used for flow cytometric analysis.

To prevent non-specific binding of antibodies to Fc receptors, cells were incubated with Fc block reagent (Biolegend, 422301) at 4°C for 15 min. Cells were then stained with FITC-conjugated goat polyclonal antibody to RSV (Invitrogen, PA1-73017) at 4°C for 30 min. Flow cytometry was performed on the BD FACSCanto (BD Bioscience), with data collected from 20,000 cells per sample. Data were analyzed using FlowJo V10 software (FLOWJO, LLC, OR). Cells were considered RSV-positive if they exhibited fluorescence more intense than that of 99.5% of the mock-infected cells.

### 2.3 Viral attachment

RSV A2 was incubated with antibodies at 37°C for 1 h, followed by addition of cells on ice for 1 h. Then, cells were collected, washed twice with PBS, fixed with 4% formaldehyde, blocked with Fc receptor block reagent, stained, and finally detected by flow cytometry.

### 2.4 Viral endocytosis

RSV A2 was incubated with antibodies at 37°C for 1 h, followed by the addition of cells and continued incubation at 37°C for 1 h. Cells were washed twice with PBS and fresh medium was added to the incubator for continued incubation for 1 h. Cells were then collected for the following assays.

Flow Cytometry: Cells were washed twice with PBS and incubated with 0.5% trypsin on ice for 10 min to remove surface-attached viruses (25). Then, Fc receptors were blocked, and intracellular staining was performed after using Fixating/Permeabilization Kit (BD Bioscience, 554714). Finally, cells were detected by flow cytometry.

TEM (Transmission Electron Microscopy): Cells were collected and centrifuged in 1.5 ml EP tubes at 1000 rpm for 5 min. The supernatant was discarded, and a fixed solution diluted 1:5 (3% glutaraldehyde: 0.1 mol/L PBS buffer) was added slowly along the wall of the tube. The cells were then resuspended and allowed to stand for 5 min at 4°C. Afterward, cells were centrifuged at 12,000 rpm for 10 min, supernatant was gently discarded, and 3% glutaraldehyde fixative was slowly added along the inner wall to avoid dispersing cells. Finally, the samples were sent to Chengdu Lilai Biotechnology Co., Ltd (Chengdu, China) for testing.

### 2.5 Viral replication

RSV A2 or rrRSV was incubated with the antibodies at 37°C for 1 h, followed by the addition of cells and continued incubation at 37°C for 1 h. Cells were washed twice with PBS and fresh medium was added to the incubator for continued incubation. Cells and supernatants were collected at 1, 6, 12, 20, 24, 36 and 48 h.

Flow Cytometry: The rrRSV-infected cells were collected, washed twice with PBS, and detected by flow cytometry for infection rates.

qRT-PCR: Total cellular RNA was extracted using an RNA rapid extraction kit (BioFlux, China). RNA was transferred to cDNA using Evo M-MLV Mix Kit with gDNA Clean for qPCR (Accurate Biology, AG11728, China). Real-time PCR was performed using the ABI PRISM 7900 HT (Applied Biosystems, Foster City, CA). The number of RSV copies was quantified with a TaqMan probe, and a standard curve of plasmid DNA was used to estimate the exact copy number of RSV N gene. Specific primers and probes for RSV A2 N gene and plasmid sequences are shown in **Table 1**.

**Table 1:** Sequences of primers.

Gene name	Direction	Primer sequence (5′–3′)
RSV A-N	F	AGATCAACTTCTGTCATCCAGCAA
	R	TTCTGCACATCATAATTAGGAGTATCAAT
Probe	–	FAM-CACCATCCAACGGAGCACAGGAGAT- BHQ1
Plasmid	–	AGATCAACTTCTGTCATCCAGCAAATACACCATCCAACGGAG CACAGGAGATAGTATTGATACTCCTAATTATGATGTGCAGAA
GAPDH	F	GTCTCCTCTGACTTCAACAGCG
	R	ACCACCCTGTTGCTGTAGCCAA
FcγRI	F	ATACAGGTGCCAGAGAGGTCTC
	R	CCAGCTTATCCTTCCACGCATG
FcγRIIa	F	ACACGCTGTTCTCATCCAAGCC
	R	GCAACAATGGCTGCTACAGCAG
FcγRIIb	F	TCCAAGCCTGTGACCATCACTG
	R	CCACTACAGCAGCAACAATGGC

TEM: This was performed as described above.

### 2.6 Viral release

Plaque assay: The supernatants were 10-fold serially diluted to a 10–^7^ dilution and added into wells of 24-well plates grown with HEp-2 cells in monolayer. After 1 h of infection, the viral solutions were removed and 1 mL of a 1:1 mixture of 0.6% agarose and 2X DMEM with 5% FBS was added. Five days post incubation, 4% paraformaldehyde was added to fix cells for 1 h. The agarose overlay was removed, the cells were stained with 0.05% neutral red, and the number of plaques was counted.

Flow Cytometry: The rrRSV supernatants (200 μL) were added into 24-wells plates grown to 80% fusion and incubated at 37°C for 2 h. Subsequently, the viral solutions were removed, fresh medium was added, and the incubation was continued for 24 h. Cells were collected for infection rates via flow cytometry.

### 2.7 RNA-seq analysis

RNA was extracted from THP-1 cells from T0, T2 and T5 groups using the RNA extraction kit (Genstone Biotech, Beijing, China). Then RNA sequencing (RNA-seq) was done by BGI Genomics Co., Ltd (Shenzhen, China). Differential expression analysis was performed using the R packag2 ‘DESEQ-2’, and genes with p < 0.05, and |log2FC| > 1 were selected as differentially expressed genes. The clusterProfiler package was used for the KEGG enrichment analysis using P < 0.05 as the statistical criteria.

### 2.8 ELISA analysis

The levels of IL-1β, IL-6, IL-10, and TNF-α in culture supernatants were measured using commercial ELISA kits (NEOBIOSCIENCE, Shenzhen, China) according to the manufacturer’s instructions.

### 2.9 FcγRs and TLR4 blocking

THP-1 cells were pre-incubated for 1 h with 20 µg/mL neutralization antibodies against TLR4 (clone HTA125) (Invitrogen,14-9917-82), or blocking antibodies (10 µg/mL) against FcγRI (10.1, BD), or FcγRIIa (BioXcell, BE0224-1MG). Then, the cells were infected to assess RSV entry rate and 12 h-infection rates.

### 2.10 Expression of FcγRs

Cells were collected 24 h after infection and subjected to real-time PCR using the previously described method. Human FcγRI, FcγRIIa and FcγRIIb levels were normalized against GAPDH for relative quantification. Specific primers for human FcγRI, FcγRIIa, FcγRIIb and GAPDH are shown in **Table 1**.

## 3 Statistical analysis

The GraphPad Prism 9.0 software (GraphPad software, San Diego California, USA) was used for data analysis. Results were expressed as the mean ± SEM (n=3), except results of RNS-Seq. Each experiment was performed 3 times; one representative experiment is depicted. One-way ANOVA was used for one-way comparisons between multiple groups, while two-way ANOVA was used for two-way comparisons between multiple groups. Differences with p values ≤ 0.05 were considered significant.

## 4 Results

### 4.1 Sub-neutralizing levels of F protein antibodies cause ADE after RSV infection via Fc-FcγR interactions

Firstly, to investigate the conditions under which ADE occurs, we used nirsevimab and palivizumab for study. Nirsevimab is an IgG1 antibody targeting the site Ø of RSV pre-F protein, while palivizumab is an IgG1 antibody targets site II of pre-F and post-F protein. Additionally, nirsevimab has a 50-fold greater neutralizing capacity than palivizumab (26).

**Figure 1:**
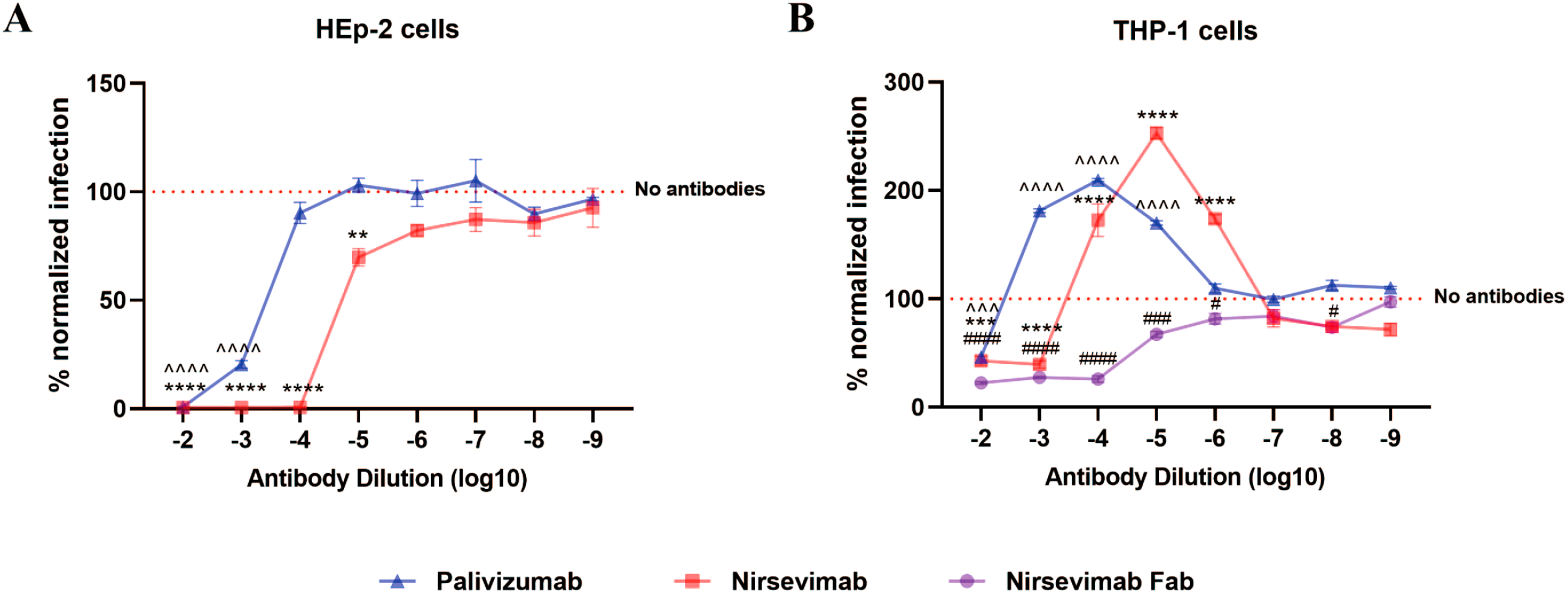
FcγRs mediate ADE of RSV infection. **(A, B)** Palivizumab, nirsevimab, nirsevimab Fab (original concentration of 1 μg/μL) at various dilutions were mixed with RSV to infect HEp-2 cells and THP-1 cells, and the infection rate for 24 h was detected by flow assay. Red dashed line represents the percentage of RSV-positive cells infected with RSV alone, against which infection was normalized. */^/# indicate significant differences between the RSV infection rates of cells treated with diluted nirsevimab/palivizumab/nirsevimab Fab and those without antibodies, respectively; # for p ≤ 0.05; ** for p ≤ 0.01; and ***, ^^^, ### for p ≤ 0.001; ****, ^^^^, #### for p ≤ 0.0001.

We mixed different dilutions of nirsevimab, nirsevimab Fab, or palivizumab with RSV, then infected HEp-2 cells and THP-1 cells. After 24 h, the percentage of RSV-positive cells was detected by flow cytometry and normalized by the infection rate in the absence of antibodies. The results showed that in FcγR-negative HEp-2 cells, both palivizumab and nirsevimab demonstrated concentration-dependent neutralization against RSV infection (**Figure 1A**). However, in FcγR-bearing THP-1 cells, enhanced RSV infection was observed at dilutions ranging from 10–^3^ to 10–^5^ for palivizumab and 10–^4^ to 10–^6^ for nirsevimab, and ADE occurred; ADE disappeared when we used nirsevimab Fab with the Fc fragment removed (**Figure 1B**). These findings suggest that FcγRs are essential for the development of ADE during RSV infection when anti-F antibodies at sub-neutralizing levels.

### 4.2 Fc-FcγR interactions increase the attachment and endocytosis of RSV

To investigate the mechanism of Fc-FcγR-mediated enhancement of RSV infection, we dissected the different steps of RSV replication in cells during ADE, which mainly include viral attachment, endocytosis, replication, and release. Experimental groups were established using HEp-2 and THP-1 cells under three conditions: RSV infection without antibodies (H0/T0), with fully protective nirsevimab concentrations (10–^2^ dilution, H2/T2), and ADE-inducing concentrations (10–^5^ dilution, H5/T5).

**Figure 2:**
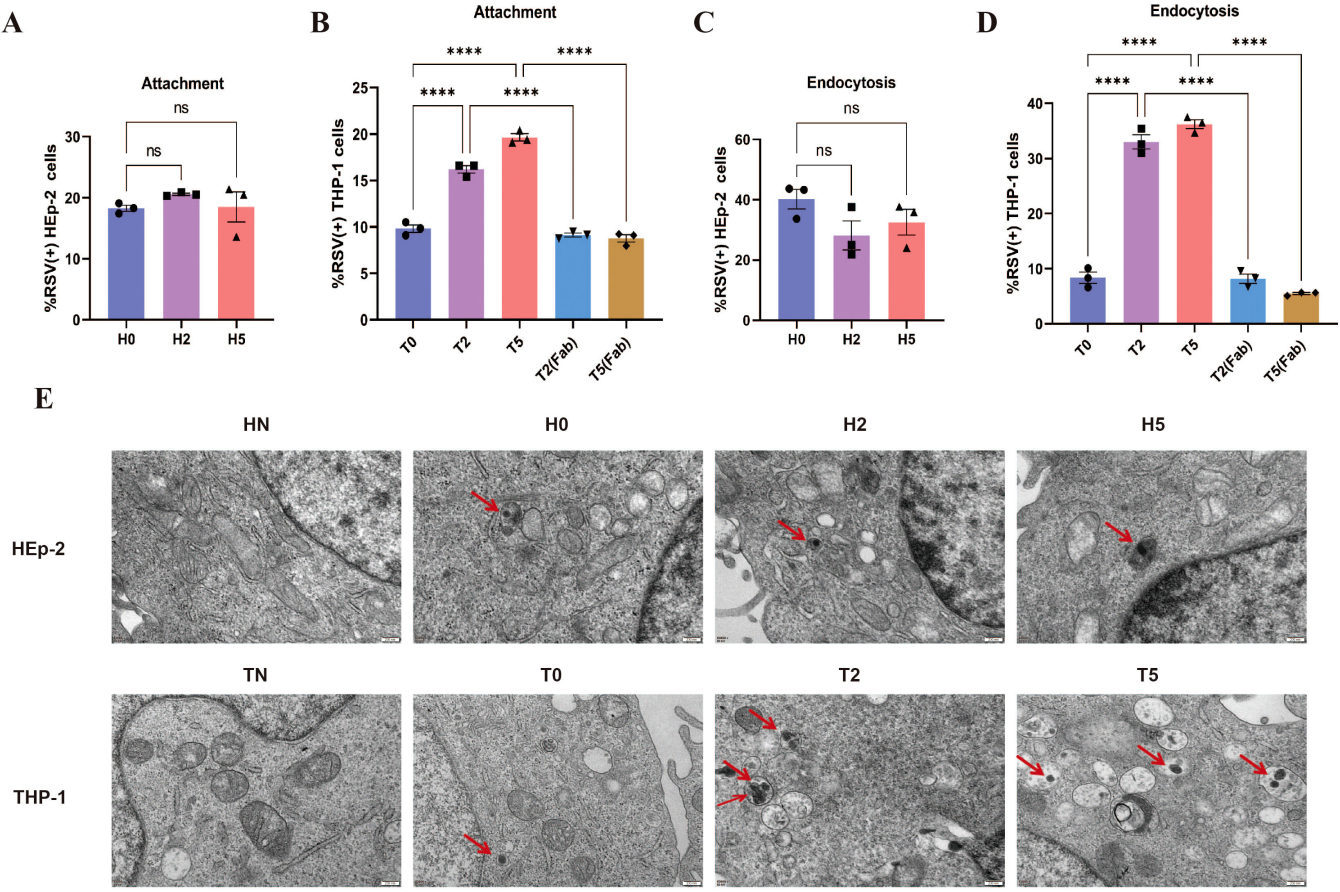
Increased attachment and endocytosis of RSV via FcγRs. **(A, B)** Attachment of RSV to the surface of HEp-2 cells and THP-1 cells by flow cytometry. **(C, D)** Endocytosis of RSV into HEp-2 cells and THP-1 cells at 1 h post-infection by flow cytometry. **(E)** TEM was used to observe the endocytosis of RSV viral particles (red arrows) into HEp-2 cells and THP-1 cells at 1 h post-infection. HN/TN, normal HEp-2 and THP- 1 cells; H0/T0, no antibody group; H2/T2, 10–^2^ dilution of nirsevimab; H5/T5, 10–^5^ dilution of nirsevimab; T2(Fab)/T5(Fab), 10–^2^ and 10–^5^ dilution of nirsevimab Fab. ns indicates no statistical significance; **** for p ≤ 0.0001.

To delineate Fc-FcγR interactions in early RSV infection, we analyzed viral attachment and endocytosis across experimental groups. Key findings revealed cell-type-specific differences. Flow cytometry showed no significant differences in RSV attachment or entry rates between antibody-treated (H2/H5) and untreated (H0) groups in HEp-2 cells (**Figures 2A, C**). However, in THP-1 cells, both T2 and T5 groups exhibited significantly elevated RSV attachment and entry rates compared to the T0 control (**Figures 2B, D**). Increase of attachment and entry disappeared when using nirsevimab Fab, which confirmed FcγRs dependence (**Figures 2B, D**). TEM allows direct observation of viral particles endocytosed into cells, and the results are consistent with flow cytometry data (**Figure 2E**). These findings indicate that F protein antibodies allow RSV attachment and entry into cells even at neutralizing concentrations, while Fc-FcγR interactions mediate additional viral attachment and entry.

### 4.3 Sub-neutralizing levels of F protein antibodies enhance RSV replication and release during ADE

**Figure 3:**
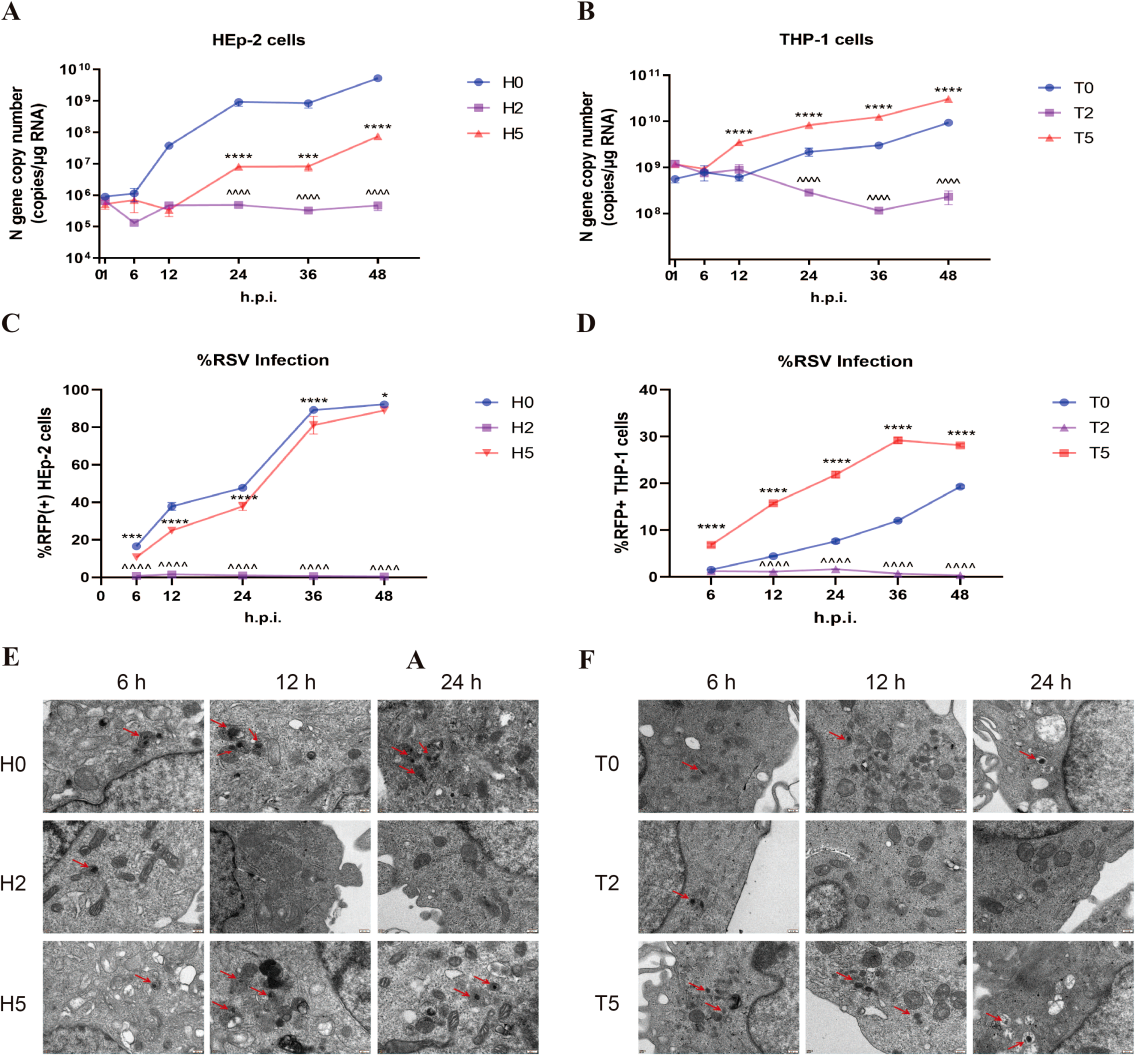
Enhanced RSV replication during ADE. **(A, B)** RSV N gene copy number was detected by qPCR in HEp-2 cells and THP-1 cells at 1, 6, 12, 24, 36, and 48h post-infection. **(C, D)** Flow cytometry was performed to detect the percentage of RFP+ cells in HEp-2 cells and THP-1 cells after 1, 6, 12, 24, 36, and 48 h of rrRSV infection. **(E, F)** TEM was used to observe RSV viral particles (red arrows) in HEp-2 cells and THP-1 cells at 6, 12, and 24 h post-infection. * indicates a significant difference between H5 and H0 or between T5 and T0 at the same time point; ^ indicates a significant difference between H2 and H0 or between T2 and T0 at the same time point. * for p ≤ 0.05; *** for p ≤ 0.001; ****, ^^^^ for p ≤ 0.0001.

To assess antibody effects on viral replication, we quantified intracellular RSV dynamics through three approaches: qPCR for N gene copy number, flow cytometry monitoring rrRSV protein translation (via RFP+ cells), and TEM visualization of viral particles. In HEp-2 cells, neutralizing antibody treatment (H2 group) induced a time-dependent reduction in N gene copy number (**Figure 3A**), near-complete suppression of viral protein synthesis (RFP+ cells ≈0%; **Figure 3C**), and TEM-confirmed viral clearance (**Figure 3E**). The above indicators gradually increased over time in the H5 group but remained consistently lower than in H0 group (**Figurse 3A, C, E**), demonstrating partial replication inhibition. However, enhanced RSV replication was observed under sub-neutralizing antibody concentrations; The N gene copy number, RFP+ cell rate, and intracellular viral particles exceeding those of the T0 group (**Figures 3B, D, F**). The results of RSV replication in the T2 group were identical to those of the H2 group. These findings reveal a dual regulation of antibodies to F protein: while neutralizing antibody concentrations universally inhibit endocytosed RSV replication across cell types, sub-neutralizing doses paradoxically enhance viral dissemination in macrophages via FcγR-mediated processes.

**Figure 4:**
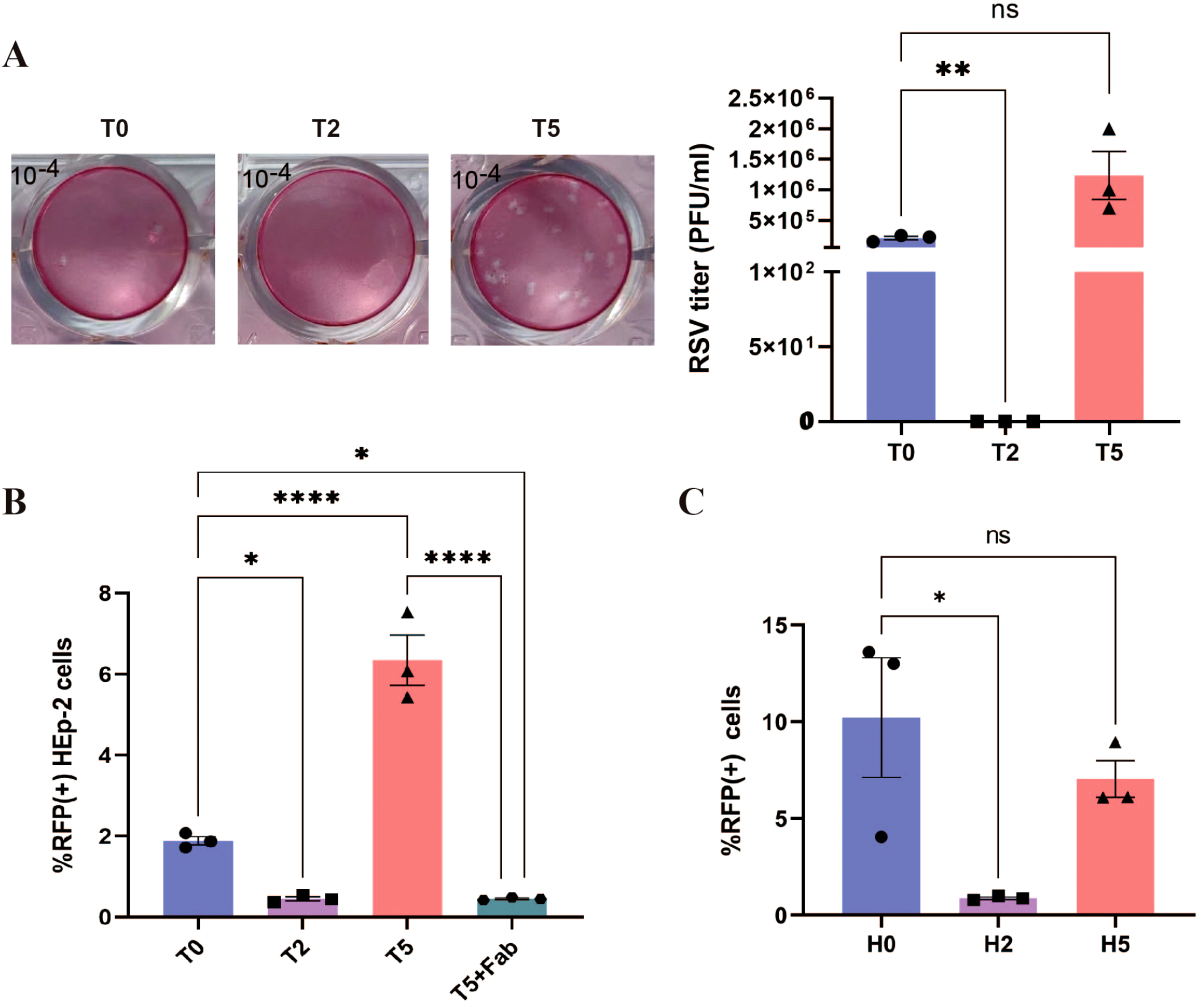
Increased RSV release during ADE. **(A)** Plaque assay was used to detect RSV titers in the supernatants of THP-1 cells at 24 h post-infection. 10^-4^: 10000-fold diluted supernatant. **(B, C)** HEp-2 cells were infected using the supernatants of rrRSV-infected HEp-2 cells and THP-1 cells at 24 h, and the percentage of RFP+ cells was detected by flow cytometry. T5+Fab: RSV was incubated with 10–^5^ dilution of nirsevimab for 1 h, followed by continued incubation with 10–^2^ dilution of nirsevimab Fab for 1 h. ns indicates no statistical significance; * for p ≤ 0.05; ** for p ≤ 0.01; **** for p ≤ 0.0001.

Subsequently, we evaluated whether antibody-enhanced infection could lead to the production of infectious viral progeny. Plaque assay revealed that the viral titer in the T5 group was 2 to 3 times higher than that in the T0 group, while no virus was detected in the T2 group (**Figure 4A**). Similarly, the infection of HEp-2 cells with the rrRSV supernatant demonstrated that the proportion of RFP+ cells was nearly zero in the T2 and H2 groups, significantly higher in the T5 group compared to the T0 group, and lower in the H5 group than in the H0 group (**Figures 4B, C**). These results suggest that the release of RSV was also increased when ADE occurs.

### 4.4 Sub-neutralizing levels of F protein antibodies seal RSV F protein incompletely to cause ADE

**Figure 5:**
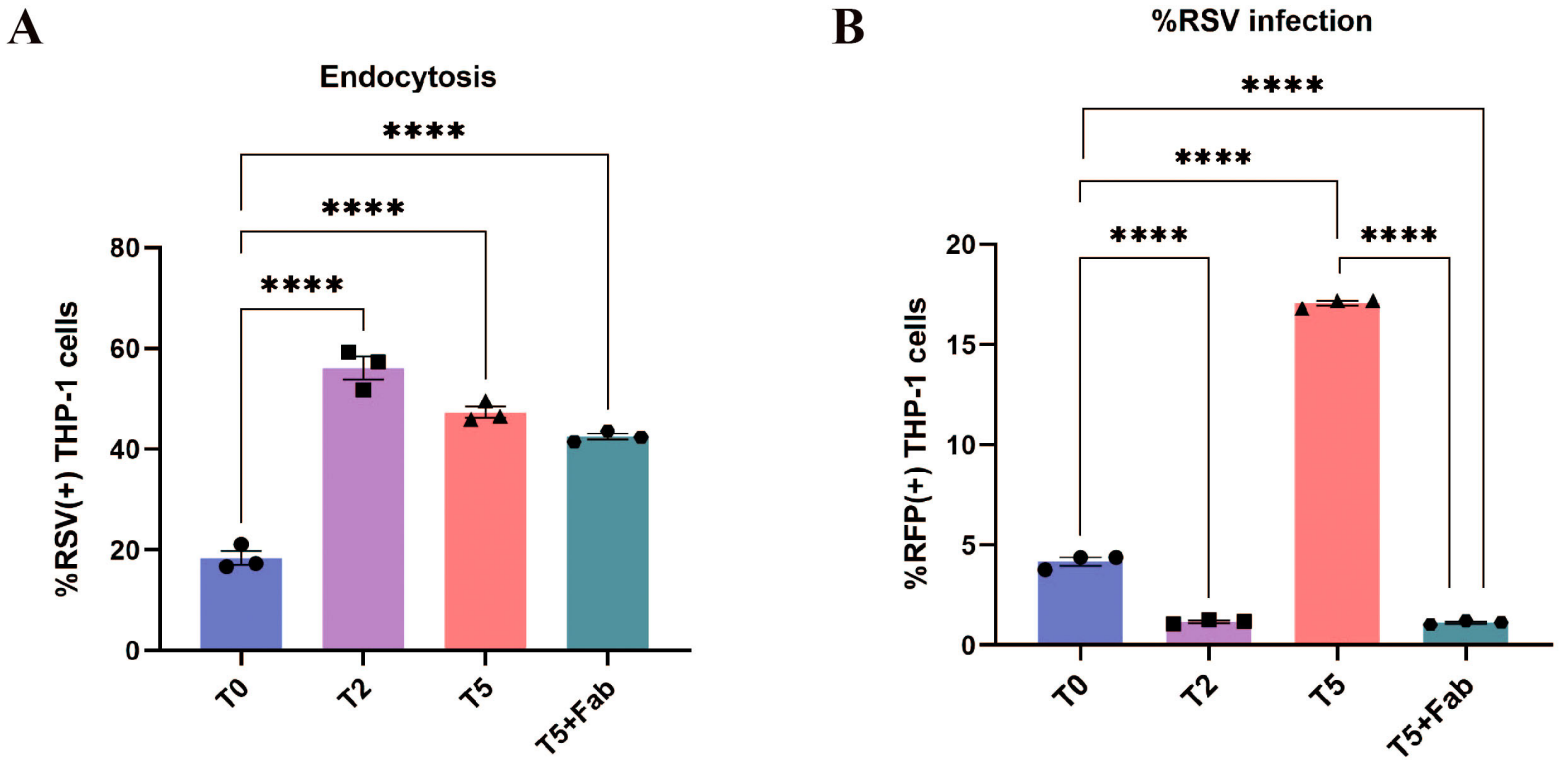
ADE disappeared after secondarily sealing the F proteins. **(A)** Endocytosis of RSV into THP-1 cells at 1 h post-infection after secondarily sealing of the F proteins by flow cytometry. **(B)** The percentage of RFP+ THP-1 cells after secondarily sealing of the F proteins was detected by flow cytometry at 24 h post-infection. **** for p ≤ 0.0001.

Our findings demonstrated that only sub-neutralizing antibody concentrations promote enhanced viral replication following RSV entry. We postulated that this effect might stem from incomplete neutralization, where sub-neutralizing antibodies fail to fully block RSV F protein-mediated membrane fusion despite facilitating increased viral entry. To test this hypothesis, we treated T5-group RSV with nirsevimab, followed by a secondary incubation with high-concentration nirsevimab Fab fragments to achieve completely F protein occlusion. The results showed that RSV entry rates post-Fab treatment remained significantly elevated compared to the T0 group (**Figure 5A**), indicating the blockade of redundant F proteins by Fab did not inhibit the internalization of the virus in THP-1 cells mediated by the interaction of Fc and FcγR. However, RFP+ THP-1 cells were virtually undetectable at 24 h post-infection (**Figure 5B**), and supernatant-reinfected HEp-2 cells similarly exhibited negligible RFP positivity (**Figure 4B**), suggesting the viral replication was inhibited. Collectively, these data demonstrate that ADE is caused by insufficient neutralization of the F protein, and that incomplete blockade of F proteins enables membrane fusion following RSV entry, thereby facilitating viral replication.

### 4.5 Exaggerated inflammatory response during ADE

**Figure 6:**
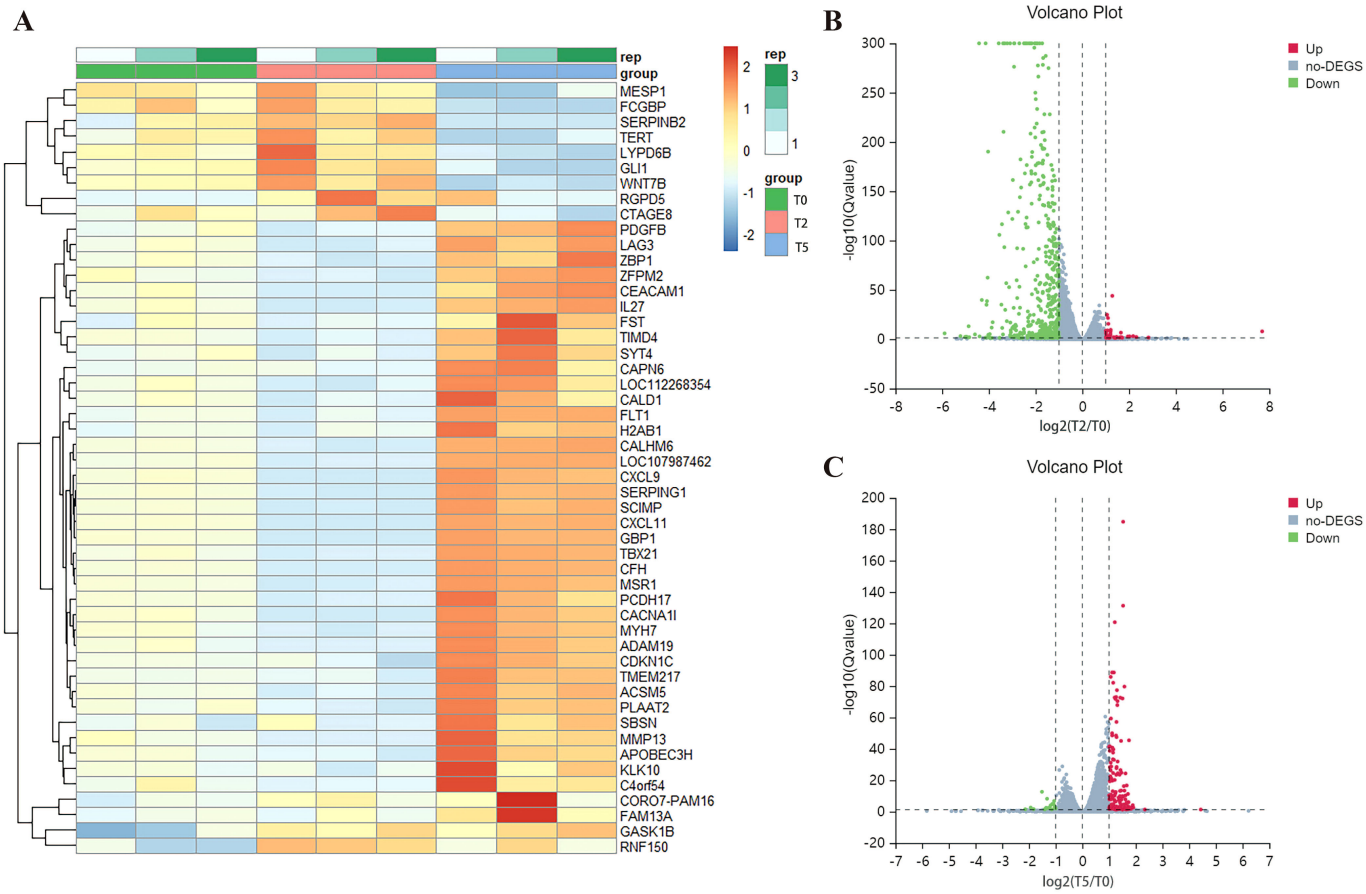
Differential gene expression analysis. **(A)** Clustering heat map of the top 50 differential genes. **(B, C)** Volcano plots of DEGs of T2 and T5 group compared with T0 group. Red represents up-regulated genes, blue represents down-regulated genes, and grey represents no significant difference genes.

To delineate the molecular changes underlying RSV ADE, we performed transcriptomic profiling via RNA sequencing (RNA-seq) on THP-1 cells from T0, T2, and T5 groups. Global gene expression analysis revealed that the majority of differentially expressed genes (DEGs) exhibiting inverse trends—genes upregulated in T2 group were downregulated in T5 group, and vice versa (top 50 DEGs visualized; **Figure 6A**). Across 16,720 analyzed genes, cells in T2 group displayed 35 significantly upregulated and 472 downregulated genes (fold change >2, P < 0.05 vs. T0; **Figure 6B**), whereas cells in T5 group exhibited 137 upregulated and 17 downregulated genes under the same thresholds (**Figure 6C**).

**Figure 7:**
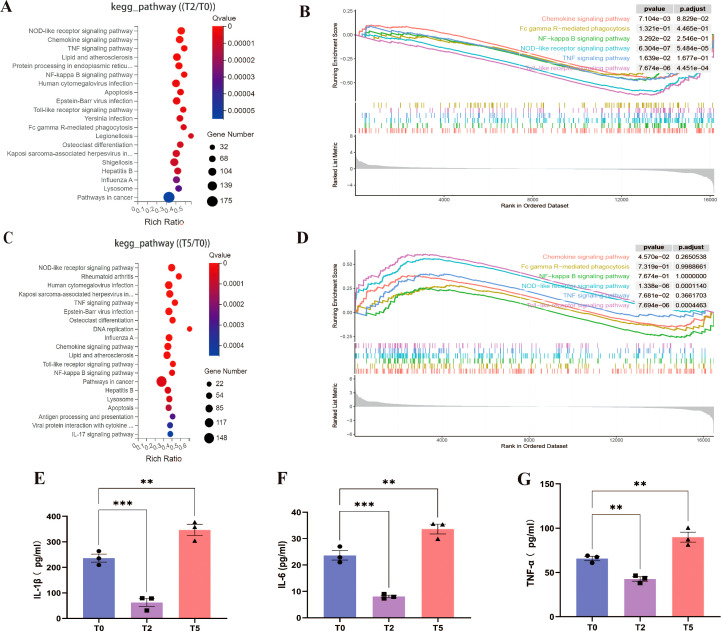
Overactivated inflammatory response during ADE. **(A, C)** KEGG pathway enrichments of T2 and T5 group compared with T0 group. **(B, D)** GSEA enrichment plots for chemokine, FcγR, NF-κB, NLR, TNF, Toll-like receptor signaling pathway of T2 and T5 group compared with T0 group. **(E-G)** Secretion of IL-1β, IL-6, and TNF-α by ELISA. ** for p ≤ 0.01; *** for p ≤ 0.001.

KEGG pathway enrichment analysis of DEGs identified the top 20 enriched signaling pathways, with immune and antiviral response pathways—including NOD-like receptor (NLR), chemokine, TNF, NF-κB, Toll-like receptor, apoptosis, and multiple virus-host interaction pathways—being significant inhibitory/activation of immune and viral response pathways in T2/T5 group (**Figures 7A-D**). Notably, the FcγR signaling pathway—a hallmark of antibody-dependent immune modulation—was prominently enriched in both groups, but was not shown in **Figure 7C** due to being ranked outside the top 20 pathways in the T5 group. These pathways collectively drive inflammatory cytokine production, prompting us to quantify IL-1β, IL-6, and TNF-α levels in cellular supernatants via ELISA. Mirroring transcriptional findings, supernatants in T5 group showed marked increases in all three pro-inflammatory cytokines, while exhibited significant suppression in T2 group relative to T0 group (**Figures 7E-G**). These results demonstrate that ADE induces a hyperinflammatory state characterized by FcγR-mediated pathway activation and dysregulated cytokine release.

### 4.6 FcγRI-TLR4 synergistic interaction and inflammatory feedforward circuit exacerbate RSV ADE

**Figure 8:**
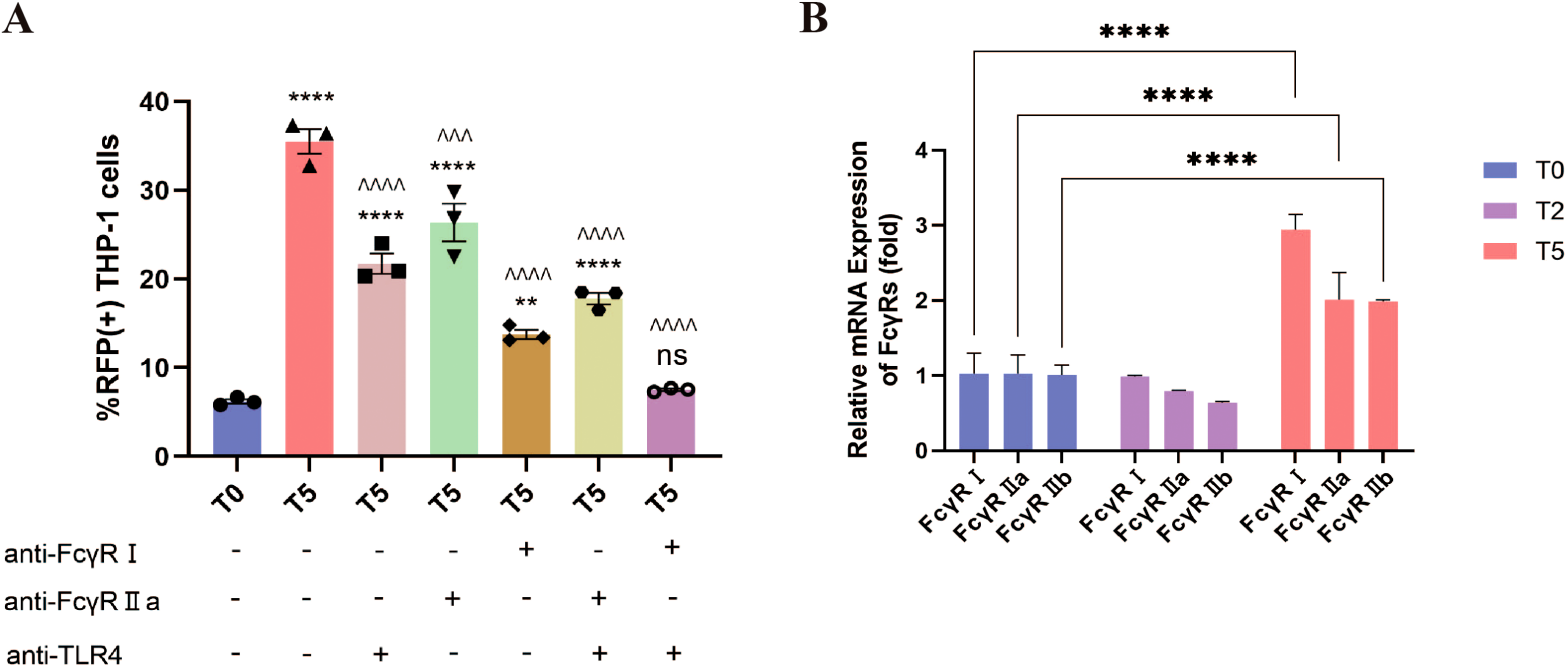
ADE requires the co-engagement of FcγRI and TLR4. **(A)** The percentage of RFP+ THP-1 cells at 12 h post-infection was detected by flow cytometry after blocking FcγRI, FcγRIIa and TLR4. **(B)** The expression of FcγRI, FcγRIIa and FcγRIIb in THP-1 cells at 24 h post-infection using qPCR. ** for p ≤ 0.01; ^^^ for p ≤ 0.001; ****, ^^^^ for p ≤ 0.0001. ns means no statistical significance compared to the T0 group.

It is reported that FcγR-mediated cytokine responses depend on activating/inhibitory receptor ratios and secondary signals like TLR activation (16, 27–30). Building on evidence implicating FcγRs in RSV ADE, we further investigated their interplay with TLR4, a known receptor of RSV F protein (31). Blockade of TLR4, FcγRI, or FcγRIIa reduced T5-group RSV infection rates, with efficacy hierarchies: FcγRI > TLR4 > FcγRIIa (**Figure 8A**). Notably, residual infection rates in all single-blockade conditions remained significantly elevated over T0 baseline. Dual blockade strategies further attenuated infectivity: concurrent inhibition of FcγRI+TLR4 reduced infection rates to T0-equivalent levels, whereas FcγRIIa+TLR4 blockade retained residual infectivity exceeding T0 (**Figure 8A**). These findings identify FcγRI as the dominant FcγR subtype driving ADE, acting synergistically with TLR4 to potentiate RSV infection.

Given evidence of inflammatory feedback regulating FcγR expression (32), we quantified FcγR transcript dynamics post-infection via qPCR. While T2-group THP-1 cells showed no significant FcγRI/FcγRIIa/FcγRIIb expression changes versus T0, T5-group cells exhibited marked upregulation of all three receptors, with FcγRI induction being most pronounced (**Figure 8B**). This FcγR upregulation, particularly FcγRI, suggests a self-reinforcing cycle: ADE-induced inflammatory signals (e.g., IL-1β/IL-6/TNF-α; **Figures 7E-G**) upregulate FcγR expression, which in turn potentiates viral entry and further cytokine dysregulation. Collectively, these data unveil a pathogenic loop wherein FcγRI-TLR4 crosstalk initiates ADE-driven viral internalization, while subsequent inflammatory cascades amplify FcγR availability, creating a feedforward circuit that exacerbates both infection and immunopathology.

## 5 Discussion

It is widely recognized that antibodies confer protection against pathogens. However, their Fc-mediated effector functions—particularly interactions with FcγRs—can paradoxically exacerbate infections via ADE in FcγR-bearing cells (15, 16). While antibody-induced RSV ADE has been observed, its molecular mechanisms remain poorly understood. Here, we systematically dissected the critical stages of ADE development during RSV infection and elucidated key mechanistic drivers.

Using a 10-fold dilution gradient of RSV antibodies in THP-1 cells, we identified ADE at sub-neutralizing concentrations (10–^3^ to 10–^5^ dilutions for palivizumab; 10–^4^ to 10–^6^ dilutions for nirsevimab). Notably, ADE was abolished when Fc-truncated nirsevimab Fab was used or in FcγR-negative HEp-2 cells, corroborating FcγR’s indispensable role (21). FcγRs recognize the Fc fragment of IgG and are expressed on myeloid cells such as monocytes, macrophages, dendritic cells (DCs), and some granulocytes (33). FcγRs, expressed on myeloid cells (monocytes, macrophages, dendritic cells), bind IgG immune complexes to mediate phagocytosis, cytotoxicity, and inflammatory responses (34).

To delineate the impact of antibodies on the lifecycle of RSV, we analyzed four viral phases (attachment, entry, replication, release) under three conditions: no antibody, protective nirsevimab (10–^2^ dilution), or ADE-inducing nirsevimab (10–^5^ dilution). RSV entry into HEp-2 cells by endocytosis and is eventually translocated into lysosomes (35), while THP-1 cells internalize antigens through phagocytosis. Both HEp-2 and THP-1 cells exhibited RSV attachment and endocytosis in the presence of antibodies, with Fc-FcγR interactions promoting these processes in THP-1 cells. However, neutralizing antibody concentrations fully inhibited replication, and enabled viral clearance; sub-neutralizing concentrations suppressed replication in HEp-2 cells but enhanced it in THP-1 cells.

RSV primarily attaches to the cell surface through interactions between its G protein and host cell adhesion molecules, with the F protein playing a secondary role in this process (36). As nirsevimab specifically targets the F protein, it does not interfere with viral attachment. Both G protein and FcγRs initiate endocytosis (10, 15, 37); however, subsequent membrane fusion—a prerequisite for cytoplasmic release of the viral ribonucleoprotein (RNP) complex (comprising L polymerase, genomic RNA, and P/M2-1/N proteins)—depends critically on F protein functionality (38). After secondary F protein occlusion using high-concentration nirsevimab Fab, we observed enhanced RSV cellular entry but abrogation of ADE. This indicates that high concentrations of antibodies completely obscure the RSV F protein, allowing viral entry but preventing membrane fusion and release of RNP, thereby elimination of viral particles. However, when antibodies are at sub-neutralizing levels, ADE occurs due to insufficient neutralization of the F protein and increased RSV entry into THP-1 cells via Fc-FcγR interactions.

How do intracellular molecular levels change when ADE occurs? Transcriptomic profiling of THP-1 cells revealed diametric gene expression patterns between T2 (protective) and T5 (ADE) groups, with predominant downregulation in T2 and upregulation in T5. Pathway analysis highlighted enrichment in inflammatory cascades (NLR, chemokine, TNF, NF-κB, Toll-like receptor, FcγR signaling). As pattern recognition receptors (PRRs), NLRs and TLRs activate proinflammatory cytokine production (39). NLR activation promotes pyroptosis via IL-1β/IL-18 release (40–42), while TLR signaling induces chemokines and cytokines (43). FcγRIIa-TLR crosstalk amplifies TNF-α, IL-1β, and IL-6 in macrophages (44), and NF-κB—a central inflammatory regulator—orchestrates IL-1β, IL-6, and TNF-α expression (45–47). These findings align with our ELISA data showing elevated IL-1β, IL-6, and TNF-α in T5 supernatants. It has been shown that IL-6 and TNF-α are signaling components of ADE (48), and elevated levels of IL-6 in RSV patients indicate a severe infection (49).

Among FcγRs, FcγRI emerged as the dominant ADE mediator: its blockade most effectively attenuated infection, while dual FcγRI/TLR4 inhibition abolished ADE. FcγRI binds to IgG with high-affinity (50), mediating antibody-dependent phagocytosis and cytotoxicity (34, 51). FcγR-mediated inflammatory responses require synergistic signals, such as TLR activation (27–30). Sub-neutralizing antibodies permit residual F protein binding to TLR4, enabling membrane fusion. Furthermore, surface expression of FcγRs is in turn regulated by cytokines. Pro-inflammatory cytokines usually increase the expression of activating FcγRs, whereas anti-inflammatory factors induce down-regulation of activated FcγRs and enhancement of FcγRIIb expression (32). Therefore, ADE-induced proinflammatory cytokines upregulated FcγR expression (particularly FcγRI), creating a self-reinforcing cycle of viral entry and inflammation.

Although RSV primarily infects respiratory epithelium, its tropism for immune cells (e.g., alveolar macrophages) in severe pediatric cases (52, 53) suggests ADE may exacerbate inflammation when antibody titers decline to sub-neutralizing levels. This FcγR-mediated immune cell infection could amplify inflammatory cascades, worsening lung pathology.

In summary, sub-neutralizing F protein antibodies enhance RSV attachment/entry via FcγR engagement, and partial F protein occlusion permits membrane fusion and RNP release, driving enhanced viral replication and ADE. Synergistic effects of FcγRI and TLR4 lead to ADE, which is enhanced by the cytokine-FcγRI feedback loop.While our *in vitro* model delineates these mechanisms, *in vivo* studies are needed to address systemic immune crosstalk. These insights may guide the development of ADE-resistant RSV antibodies and vaccines.

## Data Availability

The data presented in the study are deposited in the NCBI SRA repository, accession number PRJNA1271456.

## Supplementary material

Supplementary Figure 1 The gating strategy for flow cytometry. Cells were considered RSV-positive if they exhibited fluorescence more intense than that of 99.5% of the mock-infected cells.
