# Supplementary figures and images for: Sub-neutralizing levels of antibodies against RSV F protein enhance RSV infection via Fc-FcγR interactions
Source: Front Immunol. 2025 Jun 10;16:1594937. doi: 10.3389/fimmu.2025.1594937 (PMC12185493; doi:10.3389/fimmu.2025.1594937)

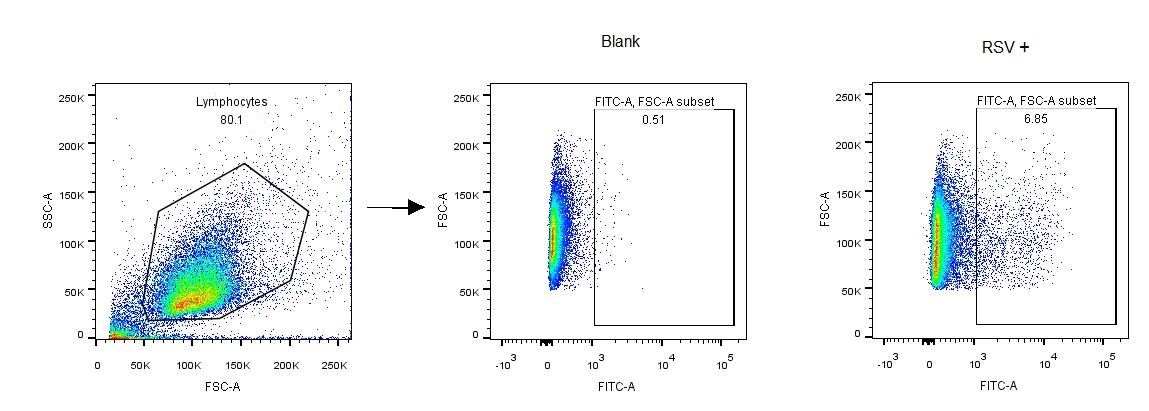

Supplement: Supplementary Figure 1 — The gating strategy for flow cytometry. Cells were considered RSV-positive if they exhibited fluorescence more intense than that of 99.5% of the mock-infected cells. [file Image1.jpeg]
